# Vitamin C modulates adrenaline-augmented gastric injury via cardiac troponin/creatine kinase pathway in Wistar rats

**DOI:** 10.22038/IJBMS.2023.68651.15019

**Published:** 2023

**Authors:** Chidiebere Ezeani, Justin Atiang Beshel, Idara Asuquo Okon, Ememfon Gabriel Inyang, Daniel Udofia Owu

**Affiliations:** 1 Department of Physiology, College of Medical Sciences, University of Calabar, Calabar Cross River State, Nigeria; 2 Department of Physiology, PAMO University of Medical Sciences, Port Harcourt, Rivers State,Nigeria

**Keywords:** Adrenaline, Anti-oxidant, Ascorbic acid, Cardiac biomarkers, Gastric juice, Myocardial injury

## Abstract

**Objective(s):**

Vitamin C has anti-oxidant benefits in the gastrointestinal tract and heart. This study investigated the effect of vitamin C on some gastric parameters in myocardial injury in rats.

**Materials and Methods:**

Thirty Wistar rats were divided into five groups (n = 6). Group 1 was the control and Group 2 (ADR) received 1 mg/kg of adrenaline subcutaneously on days 13 and 14. Group 3 received vitamin C (200 mg/kg) orally for 14 days. Group 4 received adrenaline (1 mg/kg) on days 1 and 2 and vitamin C from days 1 to 14. Group 5 received vitamin C till day 14 and adrenaline on days 13 and 14. All animals were sacrificed after 2 hr of pyloric ligation. Gastric secretion parameters were assessed while a blood sample was obtained for biochemical analysis.

**Results:**

Gastric juice volume, total gastric acidity, pepsin activity, cardiac troponin 1, creatine kinase-MB, and lactate dehydrogenase levels increased (*P<*0.05) in ADR only group relative to the control. Pre- and post-vitamin C treatment reduced (*P<*0.05) these markers to near normal. However, treatment with vitamin C reduced (*P<*0.05) ulcer score, and increased (*P<*0.05) pepsin activity, mucus weight, and serum vitamin C levels when compared with the ADR-only group. Pre-treatment with vitamin C resulted in a marked decrease (*P<*0.05) in gastric juice volume, pepsin activity, and total gastric acidity compared with post-treatment in the adrenaline-induced injury group.

**Conclusion:**

Vitamin C pretreatment reduces excessive gastric secretions, ulcer scores, and attenuates cardio-inflammatory responses in adrenaline-augmented myocardial injury in rats.

## Introduction

Myocardial injury has become a global concern due to its high mortality rate and high correlation between MI and gastrointestinal bleeding (1). Myocardial injury is due to imbalance between myocardial oxygen demand and supply (2). It may also be due to variations in myocardial oxygen supply and demand without the presence of atherosclerotic plaque or percutaneous coronary interventions and coronary artery bypass surgery (3). In these conditions, the ischemic zone is perfused giving rise to a lethal reperfusion event that is followed by increased production of reactive oxygen species and oxidative stress (4). The oxidative stress is due to excessive production of reactive oxygen species (ROS) or free radicals namely hydrogen peroxide (H_2_O_2_), radical’s superoxide (O_2_^-^), and hydroxyl radical (HO). This results in lipid peroxidation and disruption of the myocardial cell membrane (5). Endogenous anti-oxidants are mobilized to mitigate the adverse effect of ROS on the body thus resulting in anti-oxidant depletion.

Myocardial injury is responsible for several medical complications involving other vital organ failures (6). There is evidence of a link between cardiac and gastrointestinal disorders (7). And one of the organs that is greatly affected by myocardial injury with paucity of experimental evidence is the stomach (8). Acute myocardial infarction is associated with the incidence of upper gastrointestinal bleeding (9). Also, patients with acute myocardial infarction are prone to peptic ulcer disease especially when acetylsalicylic acid is administered (10). The stomach stores and modifies its gastric content for proper digestion and absorption of nutrients. Destruction of gastric mucosa due to insufficient blood flow or injury to the mucosa lining by gastric acid can lead to gastritis, indigestion, malabsorption, and malnutrition (11). Gastritis is caused by various factors including strong acidic foods, excessive alcohol, pepsin, prolonged use of non-steroidal anti-inflammatory drugs, severe infections, and stress (12, 13).

A study has shown that hormonal regulation of gastrointestinal functions is enhanced by adequate blood flow to different parts of the gastrointestinal tract including the stomach (14). Gastrointestinal dysfunction involves the stomach and the small intestine. In myocardial infarction, there is decreased blood flow to the gut and its glands accompanied by insufficient oxygen supply for proper gastro-intestinal secretions and functions (15).

Excessive release of adrenaline and increased activation of the sympathetic nervous system can elevate serum ROS levels and cause increased cardiac workload, resulting in myocardial cell injury (16), which will ultimately affect the function of other organs. Adrenaline-induced myocardial injury is an experimental model of myocardial infarction (17, 18) that is used to examine the effects of both synthetic drugs and herbs with acclaimed cardio-protective and anti-oxidant properties (19).

Vitamin C, also known as ascorbic acid, present in fruits and our daily diets is involved in many cellular functions including growth and repair of body tissues. As a potent anti-oxidant, vitamin C mitigates oxidative stress damage and enhances the production of nitric oxide, a vasodilator (20). Study has also shown that vitamin C reduces atherosclerotic plaque and prevents cardiovascular-related diseases such as hypertension and stroke (21, 22). Vitamin C also protects the heart against stress-induced heart damage and the development of cardiovascular diseases (17). It also facilitates digestion and absorption of nutrients in the gut (23). Although myocardial infarction affects gastrointestinal function, there is a paucity of information on the stomach function in this disease condition. It is hypothesized that vitamin C can protect the stomach and be beneficial in alleviating oxidative stress in conditions involving myocardial infarction. This study aimed to investigate the beneficial role of vitamin C in some gastric functions in adrenaline-induced myocardial injury in rats.

## Materials and Methods

***Chemicals and drugs***

Vitamins C, adrenaline, bovine albumin, and pentobarbitone sodium were purchased from Sigma Aldrich Chemical (St Louis, MO, USA). Rat-specific cardiac troponin I, lactate dehydrogenase, and creatinine kinases muscle/ brain kits (ELISA) were obtained from Cayman Chemical Co, USA. All other chemicals used were of analytical grade.

***Ethical Approval***

The ethical approval was obtained from the Animal Research Ethics Committee of the Faculty of Basic Medical Science, University of Calabar, Calabar, Nigeria (study approval no: 118PHY3821) and the researchers adhered strictly to the recommendations of the U.K. Animals Act (Scientific Procedures), 1986 for care and use of laboratory animal models and the University of Calabar Animal Research Ethics Committee. 

***Experimental animals and study design***

Thirty apparently male healthy Wistar rats weighing between 180 and 200 g were purchased from the animal house unit of the Department of Physiology, Faculty of Basic Medical Science, University of Calabar, Calabar, Nigeria. The rats were kept in plastic cages in a standard housing condition with a 12 hr dark/light cycle at a room temperature of 28±2 ^°^C. The animals were provided with standard rat chow and drinking water *ad libitum* and provided with nesting and adequate ventilation. The rats were divided into five (5) groups of six (6) rats each and subjected to an experimental schedule as shown below.

 Group 1: The control group was injected with normal saline only and fed rat chow and water for 14 days. 

Group 2: Adrenaline-treated group served as the myocardial injury group. Adrenaline was administered subcutaneously at a dose of 1 mg/kg body weight on two consecutive days (24) on days 13 and 14. They were fed rat chow and given drinking water for a period of 14 days. 

Group 3: Vitamin C group only. Rats were treated with vitamin C (fed orally) at a dose of 200 mg/kg (25) for 14 days. They were fed rat chow and drinking water for a period of 14 days. 

Group 4: Adrenaline-treated group with vitamin C. Rats were treated with adrenaline on days 1 and 2 followed by vitamin C (fed orally) administration, after 30 min of adrenaline injection daily for 14 days. They were fed rat chow and drinking water. 

Group 5: Vitamin C+adrenaline: Rats were pretreated with vitamin C as in group 3 from day 1 to day 14 and adrenaline was administered on days 13 and 14. The rats were fed rat chow and drinking water. After the treatment period, all animals were used for experimentation. 

***Induction of gastric ulcers by pyloric ligation, measurement pH, and collection of blood sample***

After 14 days of administration, all animals were fasted for 18 hr and were anesthetized with intraperitoneal injection of pentobarbitone sodium (35 mg/kg body weight). A small incision of 2 cm was made along the linear alba to expose the stomach. The pyloric junction was ligated using a cotton thread and the stomach was returned into its place and the abdomen was stitched back in layers. The animals were taken back to their normal cages for 2 hr during this post-operative period without food and water. All animals were sacrificed after 2 hr of pyloric ligation.

The rats were euthanized with pentobarbitone sodium (35 mg/kg) and the stomach was removed to collect the gastric juice in a beaker. After the collection of the stomach juice, it was centrifuged at 3000 rpm for 10 min, and the volume of the supernatant collected was measured. The pH of the gastric juice was measured using a pH meter. Blood samples were obtained from each rat by cardiac puncture and emptied into plain sample tubes. The blood samples were then centrifuged at 200 g for 10 min to obtain serum. The sera were stored at 4 ^°^C till further use.

***Determination of ulcer scores***

The stomach was opened along the greater curvature and was washed with normal saline, spread, and fixed on a platform to expose the gastric mucosa for a clear view. A magnifying lens and a vernier caliper were used to measure the extent of ulceration. Ulcer score was done according to the grading system as earlier reported (26) shown below: 0=no lesion (normal stomach), 0.5=pin sized ulcer, 1.0=hemorrhagic or small linear ulcer grade, 2.0=ulcer spots greater than 3 mm. The ulcer score was calculated by multiplying each grade by its frequency of occurrence. The sum of all the values formed the ulcer score for each animal.

***Measurement of total acidity, pH, and gastric mucus***

The gastric juice was titrated against 0.01N NaOH using phenolphthalein as an indicator where a pink coloration indicates an endpoint. The acidity obtained was calculated and was used to obtain the total acid output that was expressed in mEq/l using the formula according to Lee *et al*. (27). The adherent gastric mucus was measured following a previously reported method (28). After the stomach of each rat was opened along the greater curvature and pinned on a flat board, the gastric mucus was scraped off the surface of the mucosa using a spatula. It was put into a pre-weighed sterilized sample bottle containing 2 ml of distilled water. The sample bottle containing distilled water and the collected mucus were then weighed on an electronic balance. Mucus output was calculated as the difference in weights of the sample bottle containing water and the sample bottle that contained water and mucus.

***Estimation of pepsinogen activity***

The centrifuged stomach juice was used to determine pepsin enzyme activity following the modified Folin-Ciocalteau method (29). Briefly, 1 ml of bovine albumin (0.5% w/v in 0.01N HCl, pH 2) was added to gastric juice that was incubated for 20 min at 37 ^°^C. The gastric juice blank (control tube) contained only 1 ml of 0.01N HCl and was run simultaneously. Hydrolysis was stopped by adding 2 ml 0f 10% trichloroacetic acid. All the tubes were heated in a boiling water bath for 5 min and the precipitate was removed by centrifugation. A total of 1 ml of the supernatant was mixed with 0.4 ml of 2.5N NaOH and 0.1 ml of Folin-Ciocaltteu reagent, then the volume was adjusted to 10 ml using distilled water. The absorbance was measured at 680 nm. The pepsin activity was expressed as microgram per milliliter of gastric juice.

***Biochemical assay***

Vitamin C level in the serum was measured according to a known method (30). Creatinine kinases MB was measured according to the method of Zhang *et al*. (31) using an ELISA kit while serum lactate dehydrogenase activity was measured according to another method (32), using a rat specific lactate dehydrogenase ELISA kit following the manufacturer’s instruction. Cardiac troponin was measured according to the method of Mohamed *et al*. (33) using a rat-specific cardiac troponin I ELISA kit following the manufacturer’s instructions. 

***Statistical analysis***

The results obtained are expressed as mean±standard error of the mean. Data were analyzed using One-way analysis of variance (ANOVA) with the aid of the GraphPad Prism 7.0 software (GraphPad Software Inc., La Jolla, CA, USA). Where the F value was significant, Tukey’s *post-hoc *test was used to analyze differences between groups. Probability value of *P*<0.05 was considered to be statistically significant.

## Results

***Cardiac-inflammatory biomarkers and vitamin C levels***

**Figure 1:**
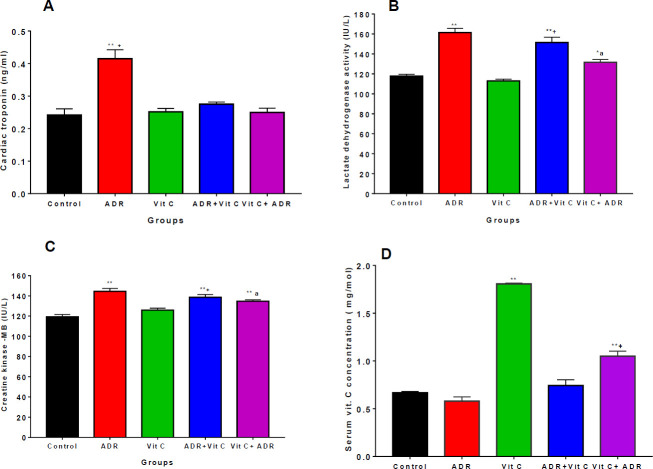
Serum of cardiac inflammatory markers and vitamin C concentration in control and ADR- treated rats

Administration of adrenaline led to significant increases (*P*<0.05) in serum cardiac troponin I (CnTn1) levels (Figure 1a), lactate dehydrogenase (Figure 1b), and muscle creatinine-kinase (CKMB) enzymes (Figure 1c) in MI only group (confirming myocardial injury) relative to the control and Vit C only groups. Treatment with vitamin C in the two treated groups (ADR+Vit C and Vit C+ADR) reduced (*P*<0.05) CnTn1 level, LDH, and CK-MB activities relative to MI-only groups tending towards the control group. The CnTn1 levels and Ck-MB activity showed no significant difference between ADR+Vit C and Vit C+ADR treated groups. These results demonstrate that vitamin C significantly reduced the markers of cardiac injury.

Our results also revealed that administration of vitamin C significantly increased serum vitamin C levels in the vitamin C only and Vit C+ADR treated groups relative to the control group and ADR only group. However, there was no significant difference between the control and ADR-only group (Figure 1d). 

***Gastric secretions***

**Figure 2:**
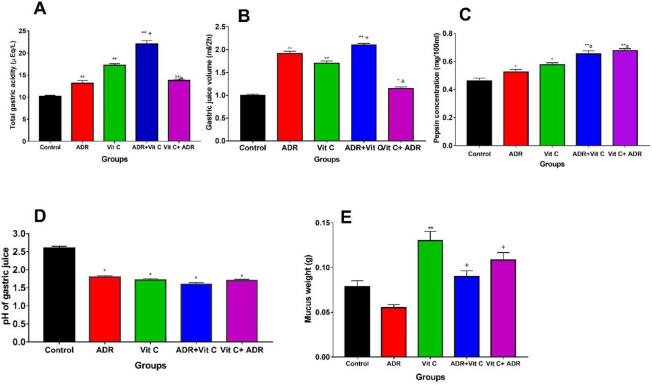
Gastric secretions parameters in control and ADR rats treated with vitamin C

The gastric secretion parameters are presented in Figure 2. The mean pepsin concentration in MI only group was significantly raised (*P*<0.05) relative to the control group (Figure 2a). The result also presented a significant (*P*<0.01) increase in pepsin activity in Vit C pre-treated (Vit C+ADR) and post-treated (ADR+Vit C) groups relative to the control and ADR-only groups. However, there was no significant difference between ADR+Vit C and Vit C+ADR groups. These results show that pepsin activity was raised in MI and vitamin C groups irrespective of the period of administration of vitamin C.

The mean volume of gastric juice produced by the stomach (Figure 2b) after 2 hr in the ADR-only group increased (*P*<0.05) relative to the control and vitamin C-only groups. The result also revealed that the volume of the gastric juice was significantly (*P*<0.01) higher in the ADR group relative to Vit C+ADR groups but reduced (*P*<0.01) relative to the ADR+Vit C group. There was a significant (*P*<0.05) decrease in the volume of gastric juice in Vit C+ADR relative to Vit C, but no significant difference was observed between the control and Vit C+ADR treated groups.

The mucus secretion was significantly (*P*<0.01) reduced in the ADR group when compared with the control and vitamin C-treated groups (Figure 2c). Administration of Vitamin C (Vit C only) increased (*P*<0.01) mucus weight relative to the control group. However, the mucus weight increased (*P*<0.01) in the Pre (Vit+ADR) and post (ADR+Vit C) treated rats with vitamin C relative to the control and ADR-only groups.

There was a significant (*P*<0.05) reduction in pH in ADR only group relative to the control group (Figure 2d). A significant (*P*<0.05) decrease in the pH of gastric juice was also recorded in the pre- and post-ADR vitamin C-treated groups (Vit C+ADR) and vitamin C-only group when compared with the control. The results show that the pH of gastric juice was raised in vitamin C and ADR -induced groups. Total gastric acidity was significantly (*P*<0.05) increased in the ADR-only group relative to the control group (Figure 2e). In animals treated with vitamin C after inducing ADR (ADR+Vit C) the total gastric acidity also increased (*P*<0.01) relative to control and ADR-only groups. However, in the pre-treated group (Vit C+ADR), there was a significant (*P*<0.01) decrease in gastric acidity relative to the vitamin C-only group but no significant change was observed relative to the ADR-only group.

***Ulcer scores in vitamin C and MI-treated rats***

**Figure 3:**
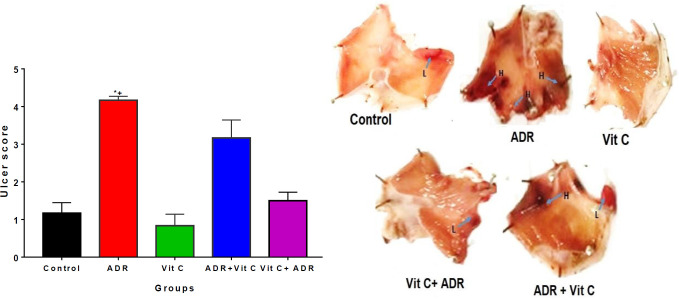
Ulcer score and macroscopic ulcer lesions in control and ADR-treated rats treated with vitamin C

The ulcer scores were increased in MI only group relative to the control group (Figure 3a). Pre (Vit C+ADR) and post (ADR+Vit C) treatment with vitamin C reduced (*P*<0.01) the mean ulcer score relative to ADR only group (Figure 3b). However, no significant difference was observed between the control and vitamin C groups.

***Weight gain in control and MI-treated rats***

**Figure 4:**
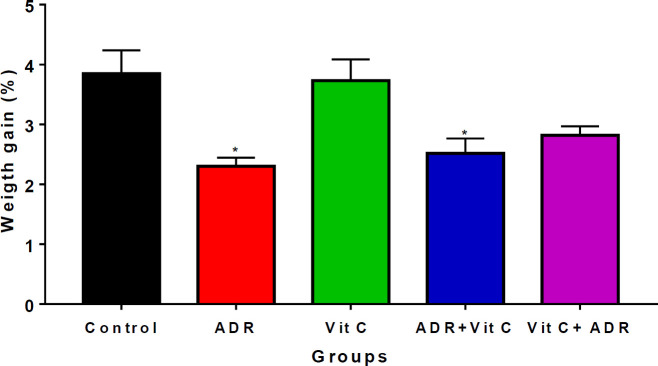
Weight gain in ADR-treated and vitamin C treated rats

The results obtained show a significant decrease (*P*<0.05) in the percentage change in the body weight of the ADR and ADR+Vit C groups compared with the control groups (Figure 4). Treatment with vitamin C significantly improved (*P*<0.05) body weight in the ADR+Vit C group in comparison with the ADR-only group. However, the percentage of body weight change following vitamin C treatment was comparable with the control. 

## Discussion

Adrenaline-induced myocardial infarction rat model (18, 24) was used for the study. This model is reliable for experimental investigation of potential cardio-protective drugs and herbs. The present study investigated the cardiac and gut regulatory response of vitamin C in adrenaline-induced myocardial injury in Wistar rats. Administration of adrenaline resulted in a significant increase in the serum concentration of cardiac troponin I (CnTn1), lactate dehydrogenase, and muscle creatinine-kinase (CK-MB) enzymes, depicting myocardial injury. Adrenaline exerts both inotropic and chronotropic effects on the myocardium. It also increases myocardial oxygen demand, and decreases blood flow to the myocardium, thereby resulting in myocardial injury (34). The serum concentration of these cardio-biomarkers is directly proportional to the integrity or severity of the myocardium (35). Their significant expression in the serum following adrenaline administration is in line with previous reports, authenticating the use of adrenaline to induce myocardial injury (18, 36). The myocardial injury consequently results in the leakage of cardiac enzymes into circulation.

Cardiac troponin I is a low-molecular-weight protein and a very sensitive and specific marker of myocardial cell injury, with blood levels rising following myocardial necrosis (37). In this study, pre-treated and post-treated animals with vitamin C after myocardial injury exhibited a significant reduction in CnTn1 levels relative to MI only group. This is consistent with a previous study (38) which found that vitamin C inhibits myocardial damage in rats by protecting the structural and functional integrity of the contractile muscles, limiting enzyme leakage from the heart into the blood.

CK-MB and LDH in serum are important diagnoses of cardiac integrity because of their increased abundance in myocardial tissue, and their virtual absence from most other tissues. The results show that MI raised serum CK-MB and LDH enzymes, which agrees with previous findings that adrenaline administration caused an increase in serum CK-MB and LDH enzymes, indicating necrotic damage to the myocardium (36). However, pre- and post-treatment with vitamin C resulted in lowered levels of CK-MB and LDH enzymes in the serum suggesting that vitamin C aids in the protection of membrane integrity, limiting the leaking of these enzymes. The result also agrees with previous reports that administration of vitamin C effectively lowered CK-MB and LDH enzymes in isoproterenol-induced myocardial infarction (38).

The mechanisms underlying the cardioprotective effect of vitamin C may be by its ability to mop up reactive oxygen species generated, preventing lipid peroxidation in the myocardium (39). As an anti-oxidant, vitamin C provides protection against oxidative stress-induced cardiac damage by scavenging reactive oxygen species produced due to increased sympathetic activity by adrenaline (40). Therefore, it is plausible that vitamin C being an anti-oxidant may help protect against oxidative cardiac injury by restricting the leakage of these enzymes from the myocardium. 

The serum level of vitamin C was significantly higher in the Vit C-only group and Vit C pre-treated group relative to the control. The decrease in serum vitamin C level in ADR and ADR +Vit C post-treated rats shows that myocardial injury might lead to the generation of oxidative stress which interferes with the mucosal protection of anti-oxidants due to increased anti-oxidant demand in the serum to cushion the effects of ROS. The decrease in serum vitamin C levels in ADR and ADR +Vit C groups is in agreement with previous studies that reported a decrease in serum vitamin C levels in myocardial injury (41, 42) perhaps due to oxidative stress and enhanced cardiac metabolism. Oral administration of vitamin C has been linked to improved anti-oxidant capacity and increased serum concentration (43).

We also investigated the effect of oral administration of vitamin C on gastric secretion parameters in adrenaline-induced myocardial injury in Wistar rats. It is well documented that adrenaline is used as a mediator of stress-induced gastric lesions and this model reproduces both local and systemic effects on the upper gastrointestinal tract resulting in bleeding gastric erosions and a decrease in mucosal blood flow (44, 45). From our results, treatment with vitamin C reduced the total gastric acidity, gastric volume, and ulcer scores with an increase in pepsin activity when compared with the ADR group. The increase in total gastric acidity in the ADR group may be attributed to the stress on the gastric mucosa membrane thereby increasing gastric secretion. It is also reported that in myocardial infarction, there is raised level of serum histamine (46) and this is a potent stimulus of gastric acid secretion. The total acidity was minimal in Vit C pre-treated rats. However, the high acidity seen in the Vit C-only group compared with the control group may be due to the acidic nature of vitamin C generally known as ascorbic acid. This is in agreement with the report that vitamin C increases gastric acidity (26, 47). The decreased gastric acid secretion in the pretreated (Vit C+ ADR) group may be beneficial in exercise or other conditions that are at risk of a sympathetic surge.

The effect of ADR on gastric secretions was occasioned by increased volume of gastric juice in ADR, vitamin C, Vit C+ ADR, and ADR +Vit C. The increase in gastric juice observed in the ADR-only group may be due to disruption of gastric mucosa due to insufficient blood flow to the stomach which ultimately resulted in secretion of fluid into the gastric lumen, thus increasing the volume of gastric juice. Experimental evidence suggests that deep damage to the mucosa may cause increased secretions into the lumen and in turn increase the volume of gastric juice (48). The decrease in the volume of gastric juice in the vitamin C pre-treated group shows that vitamin C plays a preventive role against injury to the mucosa by protecting the cytoarchitecture of the mucosa membrane (49).

Increased pepsin activity can contribute to formation of gastric ulcers (49) because pepsin solubilizes mucus, resulting in gastric mucosa ulceration (50). Our present study shows an increase in pepsin activity in ADR and Vit C pre-and post-treated groups relative to the control. The observed increase could be a result of the increased blood levels of histamine and gastrin which are documented to increase both acid and pepsin activity (51). The major event in the formation of gastric ulcers is the imbalance between the defensive factors and the offensive factors in the gastric mucosa. Defensive factors include mucin, bicarbonate ion, nitric oxide, and prostaglandins while the offensive factors are elevated secretion of gastric acid and pepsin (52, 53). Inflammation of the mucosa and oxidative stress among other factors play a key role in ulcerogenesis.

The increase in gastric injury caused by decreased blood flow to the stomach is evidenced in the level of ulcer score recorded in this study. Myocardial injury increased ulcer score in the ADR-only group, suggesting an injury to the gastric mucosa. Apart from decreased blood flow to the stomach that may result in this ulcer, the increase in ulcer score could also be attributed to increased ROS production. ROS has been linked to erosion of gastric epithelia in rats (54). Treatment with vitamin C after induction of ADR significantly decreased ulcer scores in the post-infarction-treated rats. This supports a previous report that treatment with vitamin C can reduce ulcer scores in the oxidative stress model (55).

Mucus is one of the protective mechanisms of the gastric mucosa. A decrease in mucus weight observed in ADR-induced rats showed impaired metabolic activity in gastric cells due to increased production of ROS. The increase in mucus weight in the vitamin C treatment groups is in line with the previous study of increased mucus secretion following vitamin C administration (56) and may be in part due to reducing inflammation, as well as oxidative stress, and increasing tissue oxygenation and mucus secretion in the gastric mucosa (57).

The decrease in body weight observed in ADR-induced rats is in agreement with an earlier report of decreased body weight in doxorubicin-induced myocardial toxicity (58). Adrenaline can decrease muscle mass due to increased muscle contractility and energy demand (59). Post-treatment with vitamin C improved the percentage of body weight gain, which supports the protective role of vitamin C against weight loss (60). 

There are some limitations in this study. The apoptotic markers in the heart and the stomach were not determined. The expression of apoptotic markers would have provided information on whether vitamin C has a positive influence in preventing cell death in both the heart and stomach or if it helps to regenerate the stomach cells during myocardial injury. Secondly, the histological changes in the stomach and heart were not determined. There is a need for further studies in these directions.

** significant at *P<*0.01 vs control, * significant at *P<*0.05 vs control, ^+^ significant at *P<*0.05 vs ADR; ^a^ significant at *P<*0.05 vs Vit C

Values are presented as mean ±SEM, n=6

ADR: Adrenaline

A: Total gastric acidity; B: Gastric juice volume; C: Pepsin concentration; D: pH of gastric juice; E: Mucus weight

** significant at *P<*0.05 vs control; ^a^ significant at *P<*0.05 vs Vit C, ^+^ significant at *P<*0.05 vs ADR, ^+^ significance at *P<*0.01 vs Vit C

Values are presented as mean ±SEM, n=6; ADR: Adrenaline

A: Ulcer scores; * *P<*0.05 compared with control; ^+^ *P<*0.05 compared with vitamin C and ADR induced rats treated with vitamin C; B: Macroscopic view of the stomach showing ulcers. Arrows show ulcer lesions and spots; L =Lesion; H= Ulcer spot greater than 3 mm

ADR: Adrenaline

* *P<*0.05 vs Control and ADR

ADR: Adrenaline

## Conclusion

Myocardial injury led to increased serum cardiac troponin I, creatine kinase-MB, and lactate dehydrogenase enzymes. Pre and post-treatment with vitamin C reduced these cardiac inflammatory markers to near normal. Also, treatment with vitamin C reduced ulcer score, and increased pepsin activity, mucus weight, and serum vitamin C level in both pre- and post-treated rats. However, in the pre-treatment, the pH of gastric juice was increased with a decrease in the total volume of gastric juice and total gastric acidity while the post-treated rats showed a decrease in pH of gastric juice, increased volume of gastric juice and gastric acidity, in addition to improved weight gain in the post-treated rats. From this study, it is concluded that vitamin C pretreatment can attenuate cardiac and gut inflammatory responses in adrenaline-mediated gastric injury.

## Authors’ Contributions

CE and EGI performed experiments and collected data; JAB and IAO drafted the manuscript and perform data processing; DUO designed the experiments and supervised, directed, and managed the study. CE, JAB, IAO, EGI, and DUO gave final approval for the version to be published.

## Funding

The authors declare that no funds, grants, or other support were received during the research and preparation of this manuscript. 

## Conflicts of Interest

 The authors declare no competing interests.
